# Donkey Heart Rate and Heart Rate Variability: A Scoping Review

**DOI:** 10.3390/ani13030408

**Published:** 2023-01-25

**Authors:** Marta De Santis, Samanta Seganfreddo, Alberto Greco, Simona Normando, Daniele Benedetti, Franco Mutinelli, Laura Contalbrigo

**Affiliations:** 1 National Reference Centre for Animal Assisted Interventions, Istituto Zooprofilattico Sperimentale delle Venezie, Viale dell’Università, 10, 35020 Legnaro, Italy; 2 Research Center “E. Piaggio”, Largo Lucio Lazzarino, 1, 56122 Pisa, Italy; 3 Department of Comparative Biomedicine and Food Science, Università degli Studi di Padova, Viale dell’Università, 14, 35020 Legnaro, Italy; 4 Istituto Zooprofilattico Sperimentale del Lazio e della Toscana “M. Aleandri”, 00178 Rome, Italy

**Keywords:** *Equus asinus*, HR, HRV analysis, physiological parameters, donkey

## Abstract

**Simple Summary:**

Heart rate variability (HRV) is an increasingly used research tool in animal science and is employed as a noninvasive physiological measure in animal welfare assessment, including horses. This review explores the use of heart rate (HR) and HRV in clinics and research on another, less studied, equine species: the donkey. It discusses the lack of studies and some of the technical and interpretative difficulties that can be encountered in HRV analysis in this species, highlighting the potential of this tool and the need for further studies to determine the optimal methods for its measurement and interpretation.

**Abstract:**

Heart rate (HR) and heart rate variability (HRV) are commonly used physiological measures in animals. While several studies exist on horse HRV, less information is available for donkeys. This scoping review aims to understand the extent and type of published evidence on donkey HR and HRV, their clinical and research applications, the devices used, and the analysis performed. Only quantitative primary studies published in English were considered. Four different databases were queried through the Web of Science platform, with additional evidence identified by citation chasing. After a two-stage screening phase, data were extracted considering study and population characteristics, information on HR/HRV analysis, and applications. The majority of the 87 included articles (about 80%) concerned a sample size of up to 20 individuals and were published since 2011 (about 65%). Forty-one articles employed an electronic device for signal acquisition (mainly electrocardiographs and heart rate monitors), yet only two articles reported HRV parameters. The literature on donkey HRV is lacking, and this gap can be filled by gaining knowledge on donkey characteristics and finding useful tools for welfare assessment. Comparison with what is known about the horse allows a discussion of the technical and interpretative difficulties that can be encountered with donkeys.

## 1. Introduction

Heart rate (HR) and heart rate variability (HRV) are commonly used physiological parameters in animals. While HR indicates the average number of heartbeats per minute, HRV corresponds to the fluctuation in the inter-beat intervals (IBIs) and is deemed a finer measure of the functioning of the autonomic nervous system [1,2]. In humans, HRV analysis has grown in popularity since the 1960s, thanks to the advancement of digital signal processing techniques and the discovery of its clinical relevance in fetuses and heart patients [3,4,5]. Over the last decades, the interest in HRV research (and clinical) applications has also increased in nonhuman animals, as it is considered a sensitive physiological measure that can be also registered through noninvasive equipment [1,6].

With regard to the *Equidae* family, which includes horses and donkeys, we found at least four reviews dealing with HR/HRV analysis in horses, revealing a relatively wide number of studies on the topic [1,7,8,9]. The range of applications in this species is ever-growing. HRV analysis sounds promising for the diagnosis of pathological conditions [8,10,11], for the stress assessment of riding horses in the sport and competition context [12,13], during transport [14,15] or animal-assisted interventions [16,17], and for the investigation of the horse emotional state and the mechanisms involved in human–horse interactions and relationships [9,18,19,20].

This spread of HRV analysis goes hand in hand with the advancement of technologies aimed at obtaining increasingly accurate signals using even more easy-to-wear and user-friendly devices. Wearable technologies have been also developed for horses, employing electrocardiograms (ECGs), Holter monitors, telemetric transmitters, heart rate monitors that record IBIs, and recently, photoplethysmographs. Each of these systems has its pros and cons in terms of costs, wearability, reliability, and quantity and quality of data recorded [1,8,21].

Actually, in horses, as well as in other animals, HRV analysis methodologies and data interpretation are still debated. As reported by several authors, techniques and analysis methods suffer from little standardization in the veterinary field, and the results are difficult to compare directly across studies [1,7,8]. In our opinion, the need for standardization and the many questions surrounding HRV analysis make it particularly interesting to study whether and how these physiological measures are used in different animal species. Although HRV analysis in horses is gaining popularity, from a preliminary search conducted through Web of Science and Google Scholar on another equine species, i.e., the donkey, it seems that much less information is available on the aforementioned cardiac parameters and on the way in which they are registered and used; furthermore, no reviews specifically on the topic were identified. As titled in a paper by Burden and Thiemann (2015), “donkeys are different”, their specific variations from the horse should be taken into consideration in the clinical and research contexts. These authors provided an overview of the differences between the two equine species, including behavioral, nutritional, anatomical, and physiological ones. The reported reference ranges for donkeys’ temperature, pulse, and respiration are different from those of horses, as well as their biochemical and hematological parameters [22].

Recently, donkeys are gaining popularity, partly due to the increasing variety of roles that this species plays in human society [23]. Indeed, on the one hand, donkeys are used as a workforce, as well as for milk and meat production; on the other hand, they are kept as companion animals and involved in different kinds of human–animal interactions, including animal-assisted interventions (AAIs). Therefore, it is interesting to investigate the use of HR and HRV in donkey clinics and research, especially in relation to animal welfare, where noninvasive and reliable indicators are sorely needed [23,24]. For example, it would be worthwhile to investigate whether these measures have been used to analyze the effect of potential stressors on the donkeys’ autonomic nervous system.

Hence, this scoping review had the aim of understanding the extent and type of published evidence about donkey heart rate (HR) and heart rate variability (HRV), the analysis methodologies employed, and the clinical and research application of these physiological parameters.

## 2. Materials and Methods

### 2.1. Protocol

A protocol was developed according to the JBI (Joanna Briggs Institute) methodology for scoping reviews [25] and is available upon request to the authors. The current review was written following the Preferred Reporting Items for Systematic Reviews and Meta-Analyses extension for Scoping Reviews (PRISMA-ScR) [26].

### 2.2. Eligibility Criteria

Studies reporting primary data on HR and/or HRV in donkeys *(Equus asinus)* of all ages and genders were included in this scoping review, with the exception of data on donkey embryos or fetuses, which were excluded as they go beyond the scope of this review. No geographical or date restrictions were applied to included studies. Due to time and resource limitations, only studies published in English were included. Only published peer-reviewed primary studies or short communications were considered, with no restriction regarding quantitative study designs: experimental and quasi-experimental, as well as analytical and descriptive observational, study designs were included. On the contrary, qualitative studies, reviews, books, commentaries, editorials, letters, and conference proceedings were excluded.

### 2.3. Information Sources, Search, and Selection

The search was run in January 2022 through the Web of Science (WoS) platform in the following databases: WoS Core Collection, KCI Korean Journal Database, MEDLINE^®^, and SciELO Citation Index. The search query is shown in Table 1. After checking for duplicates, one reviewer (M.D.S.) proceeded through the first step of the study selection process (title/abstract screening), discussing any doubt with a second reviewer (S.S.). The full-text screening was then carried out independently by two reviewers (M.D.S. and S.S.). Any disagreement was resolved through discussion or confrontation with an additional reviewer (L.C.). The selected full-text papers were screened through by citationchaser [27], an online tool developed for backward and forward citation chasing (August 2022). The list of references and citations was downloaded and screened by the two reviewers (M.D.S. and S.S.) to retrieve any additional source of information by applying the same eligibility criteria described in Section 2.2.

### 2.4. Data Charting and Synthesis of the Results

Full-text articles included were charted using a data extraction tool from a review by Latremouille and colleagues as a model [28]. The tool was then adapted in an iterative manner, based on the available data, for the purposes of this review. Data charting included the following sections and items: (a) characteristics of the studies (year of publication, journal, country where the study was performed, and type of publication); (b) characteristics of the population (sample size, breed/subspecies, age and weight of the sample, and sex); (c) information on HR/HRV analysis: parameters reported (HR, HR baseline, RR, i.e., R-wave-to-R-wave interval, RMSSD, i.e., root mean square of the differences between adjacent NN intervals, SDNN, i.e., SD of normal-to-normal or R-wave-to-R-wave intervals, HF, i.e., high frequency, LF, i.e., low frequency, PQRS intervals or morphology/amplitude description, and ECG trace reported) and methodologies for signal acquisition and analysis (kind of devices and analysis software used); (d) HR/HRV applications. As for applications, the framework proposed by Latremouille et al. [28] was used to classify studies into four major categories: physiological conditions; pathological conditions; responses to external stimuli; outcome predictions. Within each category, the reviewers then inductively divided the articles into subcategories. Extracted data, summarized as numbers and/or percentages, are presented through tables or graphs depending on the best graphic visualization, according to the authors, and accompanied by a narrative summary.

## 3. Results

### 3.1. Selection of Articles

**Figure 1:**
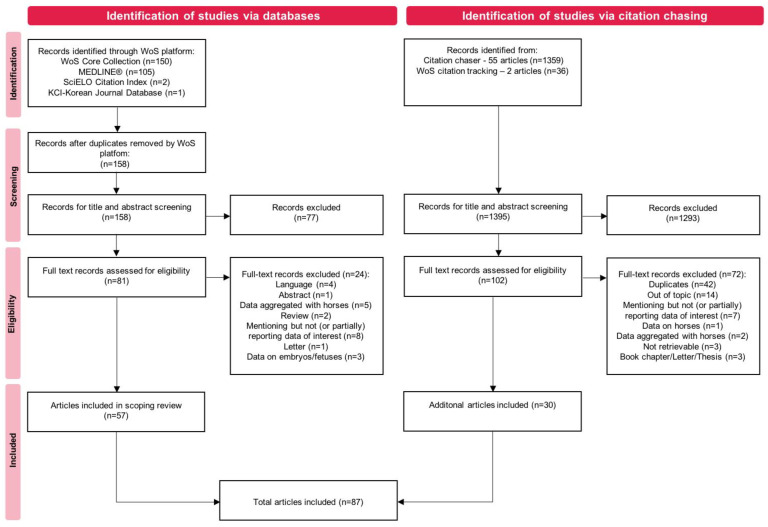
PRISMA flow diagram for record identification, screening, eligibility, and inclusion.

Of the 158 records identified via the database search, 57 were included. Backward and forward citation chasing from these 57 records resulted in the inclusion of a further 30 records, with a total number of 87 records included for data charting. The PRISMA flow diagram [26] in Figure 1 illustrates the screening process. The data charting tool with all the extracted data from each article is reported in Supplementary Materials. A graphical and narrative synthesis of extracted data is presented in the paragraphs below.

### 3.2. Characteristics of Articles

**Figure 2:**
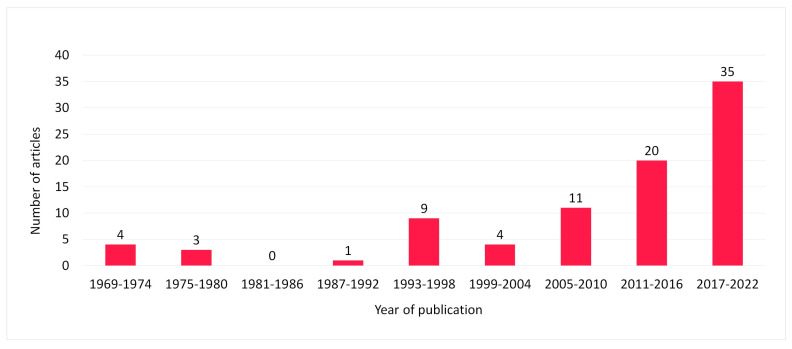
Distribution of studies published by 6 year intervals (n = 87).

**Figure 3:**
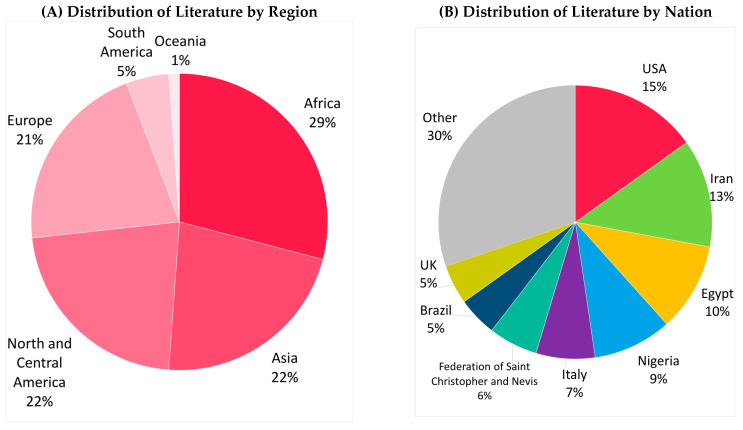
Distribution of the selected literature by region ((**A**), n = 86) and nation ((**B**), n = 86). “Other” includes Ethiopia (n = 2), Germany (n = 2), India (n = 2), Iraq (n = 2), Kenya (n = 2), Sudan (n = 2), Australia (n = 1), Austria (n = 1), Belgium (n = 1), Canada (n = 1), China (n = 1), the Czech Republic (n = 1), Jordan (n = 1), Mali (n = 1), Pakistan (n = 1), Portugal (n = 1), Saudi Arabia (n = 1), South Africa (n = 1), Spain (n = 1), and Switzerland (n = 1). One record was excluded because a country was not specified.

The included articles were published by 49 different journals, with Table 2 listing the top publishing journals. Publication dates range from 1969 to today, with 63% of articles (n = 55) that were published in the last decade (precisely from 2011), as shown in Figure 2. Most articles were from Africa (n = 25), Asia (n = 19), and North/Central America (n = 19) (Figure 3A: distribution of literature by region), with USA, Iran, and Egypt being the three countries where more studies were conducted (Figure 3B: distribution of literature by nation). Overall, 83% of articles (n = 72) were classified as original research articles, whereas the remaining (n = 15) were short communications or case reports.

### 3.3. Population

Most of the articles, about 80% (n = 68), concerned a donkey population of up to 20 individuals. Among these, studies that enrolled 5–10 individuals were the most frequent (n = 39), as shown in Table 3. The studies were also divided according to the age and weight of the donkeys involved (Table 4); most of them employed a population older than 5 years (n = 51) and weight of 100–200 kg (n = 52), although some of the studies fell into different age [29,30,31,32,33,34,35,36,37,38,39,40,41,42,43,44,45,46,47,48,49,50,51,52,53,54,55,56,57,58,59,60,61,62,63,64] or weight categories [29,30,31,33,34,36,41,42,43,45,46,47,48,53,58,62,63,65,66,67,68,69].

Two other characteristics that were taken into account to analyze the population were sex and any breed or subspecies, if mentioned, in order to characterize the sample of donkeys included in the studies. Sex was specified in most of the included articles (n = 71), and the overall ratio between the number of males and females was in favor of males (M/F ratio = 1.5), although some articles presented exclusively male [39,40,41,42,50,54,65,66,70,71,72,73,74,75,76,77,78,79,80,81,82,83,84,85,86,87] or female samples [55,88,89,90,91,92,93,94,95,96,97]. More than half of the articles (n = 52) did not specify anything about the breed or subspecies of donkeys under study, whilst the remainder enrolled Miniature (n = 11) [37,53,61,66,70,73,79,81,85,91,98], Amiata (n = 4) [63,99,100,101], Nubian or Jerusalem (n = 4) [50,71,92,102], Martina Franca (n = 3) [63,77,97], and other (Mammoth asses [58,93]; Somali [103,104]; Nordestino and North-east Brasilian [95,105]; Abyssinian [44], African [88], Dezhou [39]; Malian [75]; Mexican burro [76]; Nevisan [90]; Sardinian [101]; Zamorano-leones [43]; general feral [68], local [67], and cross [63] breeds) donkeys.

**Table 3:** Sample size of the included studies (n = 87).

Sample Size	n (%)	References
1–4	15 (17%)	[53,70,80,81,86,87,88,89,90,91,92,93,94,103,104]
5–10	39 (45%)	[31,32,33,35,36,38,41,49,50,54,58,61,64,65,66,67,69,71,72,73,74,75,76,77,82,83,84,95,96,98,101,105,106,107,108,109,110,111,112]
11–20	14 (16%)	[34,37,39,42,55,56,63,68,78,79,99,102,113,114]
21–50	10 (11%)	[29,30,45,46,48,51,57,62,85,100]
51–100	6 (7%)	[40,43,44,59,97,115]
>100	3 (3%)	[47,52,60]

n: number of articles.

**Table 4:** Age and weight categories (n = 87).

**Age Category (Years)**	**n (%)**	**References ^1^**
<1	14 (16%)	[29,30,41,51,52,58,59,60,61,72,88,91,92,100]
1–5	49 (56%)	[29,30,31,32,33,34,35,36,37,38,39,40,41,42,43,44,45,46,47,48,49,51,52,53,54,55,56,57,58,59,60,61,62,63,64,65,66,67,78,79,82,84,85,90,94,98,106,113,115]
>5–10	46 (53%)	[29,30,31,32,33,34,35,36,37,38,39,40,41,42,43,44,45,46,47,48,49,50,52,53,54,55,56,57,59,60,61,62,63,64,70,73,75,80,83,89,93,95,99,101,102,105]
>10–20	19 (22%)	[41,43,44,45,46,47,48,49,50,52,53,59,60,61,62,69,71,81,87]
>20	8 (9%)	[45,46,47,48,59,60,61,86]
Not reported/“adult”	15 (17%)	[68,74,76,77,96,97,103,104,107,108,109,110,111,112,114]
**Weight category (kg)**	**n (%)**	**References ^1^**
<100	15 (17%)	[29,30,41,42,45,53,65,66,67,72,88,92,112,113,115]
100–200	52 (60%)	[29,30,31,32,33,34,37,38,40,41,42,44,45,46,47,48,49,50,53,54,55,56,57,62,65,66,67,68,70,71,73,74,79,80,81,82,83,84,85,86,90,94,95,96,98,102,105,106,109,110,111,114]
>200–300	19 (22%)	[31,33,34,35,36,43,45,46,47,48,58,62,63,64,68,69,87,89,99]
>300	10 (11%)	[33,36,39,43,46,47,58,63,69,93]
Not reported	17 (20%)	[51,52,59,60,61,75,76,77,78,91,97,100,101,103,104,107,108]

n: number of articles; kg: kilograms. ^1^ Some studies are reported into more than one category, since the subjects enrolled fell into more than one age and weight category.

### 3.4. HR/HRV Analysis and Signal Acquisition

According to the pre-established inclusion criteria, all the studies had to report primary data on HR and/or HRV in donkeys. The HR parameter was the most prevalent, and its value was reported in all articles (n = 87). On the other hand, the parameters related to HRV were much less frequently reported, as shown in Table 5. Four other studies reported the average R–R interval, which is the interval between two R peaks of the ECG trace and corresponds to the IBI. These studies employed a Holter ECG [87] or an electrocardiograph [37,43,96] to record the signal without reporting any further HRV analysis (which could be achieved on the basis of the R–R interval time series).

Overall, the signal was acquired through different methodologies and devices, as reported by the authors: auscultation or pulse detection (n = 23) [35,36,39,40,50,51,52,58,59,65,68,71,73,74,85,90,95,98,104,105,108,110,112], electrocardiograph (n = 10) [37,41,43,45,49,76,83,89,96,103], heart rate monitor (n = 10) [29,30,32,38,64,75,78,97,101,114], multiparametric device with ECG (n = 4) [66,77,99,111], ultrasound system (n = 2) [46,48], Holter monitor (n = 2) [63,87], pulse oximeter (n = 1) [54], telemetry (n = 1) [94], or their combination (n = 11) [33,47,53,69,70,72,81,86,88,92,106]. The remaining n = 23 studies did not specify the signal acquisition methods and/or devices.

Lastly, some studies using a Holter monitor and/or electrocardiograph also reported other data that are briefly listed as follows for completeness: elements of PQRS morphology or amplitude description (n = 14) [37,43,45,53,63,70,81,86,87,88,89,92,96,106], images of the ECG trace (n = 9) [43,45,53,70,86,87,88,89,92], and PQRS intervals (n = 5) [37,43,45,89,96].

In the n = 41 articles employing various electronic devices for signal acquisition (thus excluding n = 23 studies in which HR was collected through auscultation/pulse palpation and n = 23 studies in which the device was not specified), the brand and/or model of the device used was reported in almost all cases (n = 37). Instead, the software used for analysis was specified in n = 5 articles [63,75,96,97,101], including the two articles reporting HRV parameters (Table 5). Specifically, Polar Protrainer 5 (Polar Electro Europe BV) [75] and Polar FlowSync software (Polar^®^) [101] were used for HRV analysis.

### 3.5. Application of HR/HRV Parameters

In n = 21 studies (around 24%), the parameters related to HR/HRV were reported as physiological measures collected on normal/healthy donkeys. Some of them (n = 6) were considered purely normative, i.e., reporting donkey’s HR/HRV reference or baseline values. The remaining articles, in addition to considering a sample of donkeys in physiological conditions, also examined the effect of different variables, such as age, sex, and season, on HR/HRV. These records are detailed in Table 6a. Normative/physiological HR/HRV data can be found even in other studies as resting/baseline values (n = 47) [31,32,33,34,35,36,38,39,41,42,49,54,55,56,57,58,61,64,65,66,67,69,72,73,76,78,79,82,83,84,85,95,96,98,99,101,102,103,106,107,109,110,111,112,113,114,115] and/or referring to a control group (n = 2) [85,108]. Lastly, one article concerned a population of donkeys with both physiological and pathological conditions [47].

Overall, N = 11 studies (about 13%) reported HR/HRV data in relation to a series of pathological conditions. All of these studies were case reports/short communications, as detailed in Table 6b. Some other studies implying the experimental exposure to physical, biological, or chemical potentially pathogenic agents [56,76,85,107,108] were classified in the last category, which reported HR/HRV parameters in response to external stimuli. This category consisted of n = 55 studies (63%). Most of them (n = 33) collected cardiac frequency in response to anesthesia, sedation, or analgesia, while the others considered exposure to other drugs or treatments, work and exercise (with different conditions), heat stress/dehydration, etc. A list of the applications and their references is shown in Table 6c. No studies were classified in the category of outcome predictions.

As for the HRV parameters reported in the two studies listed in Table 5, their applications were classified as follows: the RMSSD parameter was reported in two studies investigating the response to external stimuli, specifically, donkey’s response to AAI [101] and different driving methods [75]. This last study also reported other HRV parameters, namely, SDNN, HF, and LF.

## 4. Discussion

This review allowed us to explore the currently available literature on donkey HR and HRV and showed that HRV analysis is still largely unexplored in this species. The only two articles identified that reported HRV parameters [75,101] were from the past 10 years. This may be related to the fact that HRV analysis is a relatively recent field of research, especially in animals; on the other hand, most of the studies identified through this review were from the last 10 years, reflecting what McLean and Navas Gonzalez [23] already reported, i.e., that donkeys are recently gaining popularity, and that the scientific literature on this species is growing.

Looking generally at the studies reporting HR, their geographic distribution was wide, reflecting the wide distribution and versatility of donkeys (a characteristic already mentioned in Section 1). The selected studies were mainly small-scale, with a sample size of up to 20 individuals, and they showed a fair variety in terms of the age and weight ranges of the sample. It is known from the literature that individual and environmental variables can influence cardiac parameters (e.g., [29]). Table 6a shows what has been studied so far in terms of general reference values, while also considering the abovementioned variables, basically for HR. This review also made it possible to identify studies in which parameters related to donkey ECG, measured by either electrocardiographic or Holter monitors, were presented. Some of these studies were already mentioned and discussed by Mendoza and colleagues [116], who summarized the main features of donkey ECG. As commented by these authors, the different amount of information available on the cardiovascular system of horses and donkeys is also related to the differences in use between the two species. Indeed, in horses, cardiovascular diseases are frequently found during examinations due to poor sports performance. On the other hand, in donkeys, these examinations are less common, as they are less involved in riding and sporting activities [116]. Rather, 10% of the articles identified analyzed donkey performance and HR/HRV response in relation to work and exercise.

In terms of applications, HR was mainly reported—and examined—as a vital parameter in clinical cases (Table 6b) or to monitor the effect of anesthesia/sedation/analgesia or particular treatments and medications in the clinical setting (Table 6c). Compared to the horse, the donkey has peculiar characteristics, which are related to a number of evolutionary adaptations to semi-arid climates. Given the different fluid balance and water partition, pharmacokinetics differs between equine species, and anesthesia, sedation, and analgesia require species-specific protocols, as well as other anti-inflammatory drugs and antibiotics [22]. Other studies (shown in Table 6c) analyze the HR response to various potentially stressful stimuli such as work, heat, and transportation. The two studies reporting HRV were also part of this last group; the first compared HRV parameters (specifically RMSSD, SDNN, LF, and HF) in donkeys subjected to different training methods, while the second considered RMSSD before, during, and after AAI sessions. It would be interesting to further investigate these parameters as stress indicators in donkeys, comparing their responses to different stimuli. For example, they could be analyzed to assess the autonomic responses of donkeys to simple stimuli (visual, olfactory, etc.), thereby gaining more information about the perceptual and discriminatory abilities of this species. Alternatively, different management conditions or different types of training could be compared. Lastly, the effect of human–donkey interaction could be explored further, including the context of AAI, e.g., with different types of interventions and approaches to the animal.

Hence, some considerations can be made about HRV analysis in donkeys, also based on what has been reported in horses. HRV is a physiological measure that can be collected noninvasively and has several possible research applications to explore, including welfare assessment. The aforementioned peculiarities and adaptations of the donkey’s physiology affect the functioning of the autonomic nervous system, which is involved in the regulation of body homeostasis. Analysis of HRV could be pivotal to analyze its mechanisms and regulation. Each HRV parameter has its own significance in terms of SNA activation. Regarding the parameters mentioned in this review, the SDNN is influenced by both parasympathetic and sympathetic activity, RMSSD is related to vagal (parasympathetic) activity, and LF mainly reflects baroreflex activity in resting conditions, while HF is influenced by parasympathetic activity and corresponds to the variations of HR linked to respiration [2]. All of these parameters are derived from time-domain (SDNN and RMSSD) or frequency-domain (LF and HF) analysis and are among the most commonly used in the literature for the horse [1]. The analysis of HRV also allows for other time- and frequency-domain parameters to be derived, as well as nonlinear indices. However, the interpretation of these parameters is in some ways still debated, given the complex interaction that occurs between the sympathetic and parasympathetic nervous systems and other homeostatic mechanisms in the regulation of HRV [2,7].

Consequently, more studies and a shared methodology are needed to compare results. As for methods, for instance, special attention should be paid to the devices used for data collection. In horses, some critical issues in the use of heart rate monitors have been identified, and the use of ECG is recommended [1,7]. Despite this, a number of studies have compared data obtained from different heart rate monitors and ECG to assess the reliability of these devices [117,118,119,120]. It would be helpful to also carry out this comparison with donkeys. Donkeys have wider subcutaneous fat and thicker skin than horses [116], and these factors, along with their dense fur, can pose an obstacle IBI detection. In addition, there are a number of further issues related to HRV analysis to consider, as revealed in the horse literature. To name a few, frequency band thresholds for HRV analysis are species-specific [1,7], and, to our knowledge, they have not yet been determined in the donkey. Moreover, the length of recordings and the degree of artefact correction seem to play a crucial role [8,120], and these aspects also need to be taken into account in HRV analysis. In particular, as discussed by Broux and colleagues [11], several software programs are already used for HRV analysis in horses (some of which are simpler to use and others of which are more complex). However, each of these programs uses its own, generally unknown, QRS detection algorithm and type of filtering that may affect HRV analysis and, thus, the results obtained. In order to limit these issues related to the heterogeneity of study methodologies, as Latremouille and colleagues [28] concluded in their review of human neonatal HRV, consistency in reporting can be a first step, including information on the devices and analysis software used, data handling, and calculated parameters.

### Limitations of the Study

Although our review followed a rigorous methodology, we identified some weak points. Firstly, the initial search was designed with a narrow breadth, but this limitation was compensated for (at least partially) by the citation chasing phase, which allowed additional records to be identified. In addition, books and abstracts were excluded from the search, although they could have provided more data. However, we decided to focus our search on scientific papers because they undergo a peer-review process that makes the reported data more reliable. Moreover, the first screening phase (title and abstract screening) was carried out by only one reviewer, although any doubts were discussed with the other two reviewers.

## 5. Conclusions

This scoping review allowed us to analyze the existing literature in relation to donkey HR and HRV. It enabled us to highlight how, compared to the horse, the literature on HRV in the donkey is lacking; this gap can be filled in order to learn more about the characteristics of the species and find useful tools for welfare assessment. Similarly, the comparison with what is known for the horse allowed us to present some of the technical and interpretative difficulties that may also be found in the donkey. Lastly, this review can be a useful tool to find information on studies related to HR and HRV and their applications in the *Equus asinus* species.

## Figures and Tables

**Table 1:** Search query formulated for Web of Science platform.

Web of Science (All Databases Option):
(TS = ((heart rate OR heart rate variability OR HRV OR HR or IBI OR telemetry OR electrocardio* OR heart rate monitor OR Holter OR echocardio*))) AND TS = ((donkey OR equus asinus))

**Table 2:** Top journal publishing three or more articles on the topic of interest of this review (n = 87).

Journal Name	n (%)
Journal of Equine Veterinary Science	19 (22%)
Veterinary Anaesthesia and Analgesia	5 (6%)
Equine Veterinary Journal	4 (5%)
Equine Veterinary Education	3 (3%)
Veterinary Record	3 (3%)

n: number of articles.

**Table 5:** HRV analysis methods (n = 87).

HRV Parameters	n (%)	Kind of Device Used [Reference]
RMSSD (time-domain analysis)	2 (2%)	Heart rate monitor [75,101]
SDNN (time-domain analysis)	1 (1%)	Heart rate monitor [75]
HF (frequency-domain analysis)	1 (1%)	Heart rate monitor [75]
LF (frequency-domain analysis)	1 (1%)	Heart rate monitor [75]

n: number of articles.

**Table 6:** HR/HRV applications (n = 87).

**(a) Physiological Condition**	**n (%)**	**References ^1^**
Normative	6 (7%)	[40,45,46,51,62,89]
Age effect	6 (7%)	[29,30,37,44,52,60]
Sex effect	7 (8%)	[37,43,47,48,52,60,100]
Season/environmental variables effect	5 (6%)	[29,37,50,71,105]
Time of the day/diurnal rhythm effect	8 (9%)	[29,30,50,52,63,71,94,105]
Other effects	9 (10%)	Individual variation [50]; activity/workload [44]; fasting/feeding [94]; respiratory rate [47,52]; body temperature [52]; M-mode or 2D variables [48]; different Apgar scores [100]; weight/BCS [44].
**(b) Pathological conditions**	**n (%)**	**References ^1^**
Heart disease with pacemaker implantation	4 (5%)	[53,81,88,92]
Intoxication/toxicosis	2 (2%)	[70,87]
Other	6 (7%)	Cardiovascular [47]; congenital disease [91]; mastitis [93]; preputial sarcoid [80]; pulmonary fibrosis [86]; suspected mitral valve dysplasia [90]
**(c) Response to external stimuli**	**n (%)**	**References ^1^**
Anesthesia/sedation/analgesia	33 (38%)	[31,33,34,35,36,41,42,49,54,55,57,58,65,66,67,68,69,72,73,74,77,82,83,84,95,98,99,104,106,109,111,112,113]
Other drugs or treatments	8 (9%)	Anti-inflammatory [79,85]; calcium-channel blocker [96]; experimental infection with *Trypanosoma brucei* [108]; radiation [76]; vaccination with or without radiation [107]; induced endotoxaemia [56]; pycnogenol [78]
Work and exercise	9 (10%)	[32,38,39,64,75,78,102,114,115]
Heat stress/dehydration	3 (3%)	[59,102,103]
Other	4 (5%)	AAI [101]; different habituation protocols for the milking parlor [97]; transportation [61,110]

n: number of articles. ^1^ Some studies are reported in more than one category.

## Data Availability

Not applicable.

## Supplementary Materials

The following supporting information can be downloaded at https://www.mdpi.com/article/10.3390/ani13030408/s1: Table S1. Data charting of included articles.

## Abbreviations

The following abbreviations are used in this manuscript:

AAI	animal-assisted intervention
ECG	electrocardiogram
HR	heart rate
HRV	heart rate variability
IBI	inter-beat interval
JBI	Joanna Briggs Institute
PRISMA-ScR	Preferred Reporting Items for Systematic Reviews and Meta-Analyses extension for Scoping Reviews
RR	R-wave-to-R-wave interval
RMSSD	root mean square of the differences between adjacent NN intervals
SDNN	standard deviation of normal-to-normal or R-wave-to-R-wave intervals
HF	high frequency
LF	low frequency
WoS	Web of Science
